# An Exploratory Analysis of Changes in Mental Wellbeing Following Curcumin and Fish Oil Supplementation in Middle-Aged and Older Adults

**DOI:** 10.3390/nu12102902

**Published:** 2020-09-23

**Authors:** Julia C. Kuszewski, Peter R. C. Howe, Rachel H. X. Wong

**Affiliations:** 1 Clinical Nutrition Research Centre, School of Biomedical Sciences and Pharmacy, University of Newcastle, Callaghan 2308, Australia; Julia.kuszewski@uon.edu.au (J.C.K.); Rachel.wong@newcastle.edu.au (R.H.X.W.); 2 Institute for Resilient Regions, University of Southern Queensland, Springfield Central 4300, Australia; 3 UniSA Allied Health & Human Performance, University of South Australia, Adelaide 5000, Australia

**Keywords:** curcumin, fish oil, mood, subjective memory complaints, APOE4, randomized controlled trial

## Abstract

Curcumin has previously been shown to enhance mood in non-depressed older adults. However, observed benefits were limited to short-term supplementation (4 weeks). In a 16 week randomized, double-blind, placebo-controlled, 2 × 2 factorial design trial, we supplemented overweight or obese non-depressed adults (50–80 years) with curcumin (160 mg/day), fish oil (2000 mg docosahexaenoic acid +400 mg eicosapentaenoic acid/day), or a combination of both. Secondary outcomes included mental wellbeing measures (mood states and subjective memory complaints (SMCs)) and quality of life (QoL). Furthermore, plasma apolipoprotein E4 (APOE4) was measured to determine whether APOE4 status influences responses to fish oil. Curcumin improved vigour (*p* = 0.044) compared to placebo and reduced SMCs compared to no curcumin treatment (*p* = 0.038). Fish oil did not affect any mood states, SMCs or QoL; however, responses to fish oil were affected by APOE4 status. In APOE4 non-carriers, fish oil increased vigour (*p* = 0.030) and reduced total mood disturbances (*p* = 0.048) compared to placebo. Improvements in mental wellbeing were correlated with increased QoL. Combining curcumin with fish oil did not result in additive effects. This exploratory analysis indicates that regular supplementation with either curcumin or fish oil (limited to APOE4 non-carriers) has the potential to improve some aspects of mental wellbeing in association with better QoL.

## 1. Introduction

Approximately 10–20% of older adults worldwide are affected by late-life depression, defined as a major depressive episode after the age of 60 [1,2]. Unfortunately, depression often goes undetected in the elderly due to individuals under-reporting their symptoms and symptoms being confused with other age-related issues by family members or health care workers [3]. Consequently, depression is often left untreated, which in turn can lead to poor quality of life (QoL) and an increase in morbidity, disability and dependence [1]. Poor mood has also been shown to be closely associated with subjective memory complaints (SMCs), which are considered to reflect early cognitive changes and to increase a person’s risk to progress to mild cognitive impairments or dementia [4,5,6,7]. One potential preventative strategy to reduce the risk of late-life depression is to supplement the diet with mood-enhancing bioactive nutrients, such as long-chain omega-3 fatty acids (LCn-3 PUFAs) and curcumin, to improve mood in order to counteract development of depressive symptoms and poor mental wellbeing.

Curcumin, the main active polyphenolic compound of the curry spice turmeric (*Curcuma longa*), has been shown to reduce depressive symptoms in individuals suffering from depression [8] and, more recently, to improve mood in non-depressed healthy older adults [9,10]. In 2015, Cox et al. showed that curcumin supplementation (80 mg per day) for four weeks significantly reduced fatigue and attenuated negative effects of a cognitive test battery on calmness and contentedness [9]. In a partial replication study, Cox et al. extended the curcumin supplementation and measured outcomes at four and twelve weeks [10]. Again, fatigue was shown to be reduced following four weeks as well as twelve weeks of supplementation. Furthermore, curcumin significantly reduced tension, anger, confusion and total mood disturbance. However, these beneficial effects were only found following 4 weeks of supplementation. Combining curcumin with other bioactive nutrients known to counteract depressive symptoms, such as the LCn-3 PUFAs found in fish/seafood and fish oil, could be a potential strategy to extend the mood-enhancing effects over longer periods due to potential additive or synergistic effects of the combination [11].

A large body of epidemiological and observational studies shows an inverse association between fish intake and the prevalence of depression and that depressed adults have lower blood and adipose tissue levels of LCn-3 PUFAs [12,13]. This suggests that increasing one’s Omega-3 Index with fish oil supplementation might help to counter depression. This has, however, proven difficult to confirm in clinical trials, as they were mostly focused on people with clinical depression. Nevertheless, the majority of these studies showed that fish oil supplementation can reduce depressive symptoms in a variety of populations, including older adults [14,15,16]. Only a limited number of studies has examined the potential of fish oil to prevent the risk of depression in mentally healthy older adults by enhancing their mood, with mixed results. Additionally, these studies focused on depressive symptoms only, but did not measure fish oil’s effects on other mood states [17,18,19]. This indicates a need for further investigation, which should also take the apolipoprotein E4 (APOE4) status of participants into account, since the e4 variant of APOE has been shown to influence the effects of fish oil [20,21,22]. However, it is unknown whether APOE4 status influences the effects of fish oil on mental wellbeing measures. In contrast, response to curcumin supplementation seems to be unaffected by APOE4 status [23].

We recently reported the independent and combined effects of fish oil and curcumin supplementation for 16 weeks on systemic and cerebrovascular function (primary outcome cerebrovascular responsiveness (CVR) to hypercapnia) in overweight or obese middle-aged and older adults with a sedentary lifestyle [24]. In the same study, we also examined effects on mental wellbeing measures (mood states and SMCs) and general health perception (QoL) and whether the response to fish oil might be influenced by a participant’s APOE4 status. The aim of this exploratory analysis was to (1) confirm the mood-enhancing benefits of curcumin reported by Cox et al. and determine whether combining curcumin with fish oil would result in additional, longer-lasting benefits on mood states, as well as improvements in SMCs and QoL; and (2) investigate the independent effects of fish oil supplementation on mental wellbeing measures and QoL and whether they are affected by APOE4 status.

## 2. Materials and Methods

### 2.1. Study Design and Population

Community-dwelling adults residing in the Hunter region of New South Wales, Australia, were recruited to participate in a 16 week randomized, double-blind, 2 × 2 factorial, placebo-controlled intervention trial. Volunteers were eligible if they were aged between 50 and 80 years, were overweight or obesity (body mass index (BMI) 25–40 kg/m^2^) and had a sedentary lifestyle (<150 min of planned physical activity per week). Volunteers were excluded if they had an average fish/seafood intake above two serves per week or more than 300 mg/day of LCn-3PUFA from fish oil supplements, had suspected dementia (<82/100 points on Addenbrooke’s Cognitive Examination III, determined during first screening visit), were diagnosed with major depression (current diagnosis), had a history of cardiovascular, kidney or liver disease or neurological condition, or were currently on insulin or warfarin therapy. The trial was conducted at the University of Newcastle’s Clinical Nutrition Research Centre in accordance with the International Conference on Harmonization Guidelines for Good Clinical Practice. This study was approved by the University of Newcastle’s Human Research Ethics Committee (H-2016-0170) and registered with the Australian and New Zealand Clinical Trials Register (ACTRN12616000732482p). Written consent was obtained from each participant prior to commencement.

### 2.2. Study Procedures

Eligible participants attended the research facility for a total of four visits—two at the beginning and two at the end of the intervention. The secondary outcomes described in this manuscript were obtained during the second and fourth visit. During these visits, participants had fasted overnight (at least eight h) for collection of a fasting venous blood sample (2 × 10 mL) by a trained phlebotomist at a commercial pathology centre. One sample was used for routine analysis of cardiometabolic and inflammatory markers in serum and the other was centrifuged to separate plasma from red blood cells and respective aliquots were kept for further analysis of the Omega-3 Index (eicosapentaenoic acid (EPA) and docosahexaenoic acid (DHA) concentrations in erythrocyte membranes) [24] and plasma APOE4 concentrations.

After blood collection, participants were offered water and snacks before filling in questionnaires about their mental wellbeing and QoL. Mental wellbeing measures included mood states and SMCs. Measures were repeated in the same order at the end of the intervention. Furthermore, in order to assess participants’ depressive symptoms at baseline, the Centre for Epidemiologic Studies Depression Scale (CES-D) questionnaire [25] was administered. A score above 16 out of 60 points indicates a risk of depression.

#### 2.2.1. Mood States

The Profile of Mood States (POMS) questionnaire, containing 65 descriptive words, was used to assess participant’s various mood states over the last seven days before their scheduled visit [26]. It contains six mood subscales, included tension-anxiety, depression, anger-hostility, fatigue, confusion-bewilderment and vigour, which were then expressed as percentages of their maximum score for each subscale. Total mood disturbance (TMD) was calculated by averaging the percentages for all negative mood subscales and then subtracting the percentage obtained for vigour. Values ranged from −100%, indicating low mood disturbance, to +100%, indicating high mood disturbance.

#### 2.2.2. Subjective Memory Complaints

SMCs were assessed using a 27-item ‘yes’ or ‘no’ questionnaire, which is a self-assessment of memory complaints. The first three questions were used to determine whether participants had any subjective memory complaints: “Do you perceive any memory or cognitive difficulties?”, “Would you ask a doctor about these difficulties?” and “In the last two years, has your memory or cognition declined?”. The remaining 24 questions were more specific, relating to difficulties in remembering conversations/appointments/names/recent news, concentration problems, difficulties starting or keeping track of conversations and difficulties keeping track with daily activities due to any decline in memory over the past two years. The positive responses (‘yes’) were summed and expressed as a percentage of the maximum score (24 points).

#### 2.2.3. General Health Perception (Quality of Life)

The 36-Item Short-Form Survey (SF-36) was used to measure participants’ perception of physical and mental wellbeing in the last four weeks before their scheduled visit and reflects QoL [27,28]. It includes eight subscales (physical functioning, body pain, role of physical limitation, general health perceptions (=physical wellbeing subcomponent), social functioning, general mental health, role of mental limitation and energy/vitality (=mental wellbeing subcomponent), each with a maximum score of 100, indicating no disability. An average of all subscales yielded the overall QoL score.

#### 2.2.4. APOE4 Analysis

Only plasma aliquots from participants who had been allocated to one of the fish oil treatments (fish oil alone or in combination with curcumin, *n* = 65) were used to measure APOE4 concentrations, since APOE4 status has previously been shown to influence responses to fish oil [20] but does not appear to influence responses to curcumin supplementation [23]. APOE4 analysis was performed at the Hunter Medical Research Institute by a trained researcher who was blind to the intervention procedures. Plasma APOE4 concentrations were measured using a commercial Apolipoprotein E4 (human) Enzyme-linked Immunosorbent Assay (ELISA) kit (Biovision, Milpitas, CA, USA). Sensitivity of the assay was 25 ng/mL with a detection range of 50–800 ng/mL, 8% intra-assay reproducibility and 12% inter-assay reproducibility, as reported by the manufacturer. Participants with plasma APOE4 concentrations that fell within the detection range were deemed to be APOE4 carriers, i.e., with an APOE2/E4, APOE3/E4 or APOE4/E4 genotype.

### 2.3. Investigational Product and Intervention

The intervention supplements were supplied by Blackmores Institute (Sydney, Australia) and were identical in appearance to their respective placebos, identifiable only by code. Participants were allocated to one of the four treatment groups by an independent investigator according to Altman’s allocation by minimization method [29] based on their age, BMI and sex:

- FO group: active fish oil capsules (Blackmores Omega Brain™: 400 mg EPA and 2000 mg DHA/day) with placebo curcumin capsules (maltodextrin with yellow food colouring);
- CUR group: active curcumin capsules (Blackmores Brain Active™: 800 mg Longvida^®^ containing 160 mg curcumin/day) with placebo fish oil capsules (mix of corn and olive oil with 20 mg of fish oil to match odour);
- FO + CUR group: active fish oil and active curcumin capsules;
- PL group: placebo fish oil and placebo curcumin capsules.

Participants were instructed to consume six capsules daily, two fish oil and one curcumin (or matching placebos) in the morning and again in the evening with meals, and to record their supplement intake in an assigned diary, together with any changes in medication intake. The curcumin supplement was identical to that used in the studies by Cox et al. [9,10]. However, as our participants were supplemented twice a day, their total dose was double that used previously. The twice daily supplementation schedule was an attempt to increase curcumin’s efficacy by ensuring a sustained level in the blood, as curcumin has a relatively short half-life (approx. 7.5 h for Longvida^®^ curcumin [30]). For fish oil, splitting the dose into twice per day can also help to minimize fishy burps, which are commonly reported as an unpleasant side effect. The fish oil dose was based on previous literature indicating the need for high DHA doses to see effects [18,31,32].

Participants were further instructed to maintain their habitual diet and exercise regimen. At mid-intervention, participants were followed up with a phone call to enquire about their wellbeing. At the end of the trial, participants returned any remaining supplements. Capsule counts and changes in erythrocyte Omega-3 Index (analysed as described in Kuszewski et al. [24]) were used to monitor overall compliance. Blinding was maintained until all data analysis had been completed.

### 2.4. Statistical Analysis

This is an analysis of secondary outcomes from our previously published clinical trial [24]. An estimated 136 participants were needed to detect a 0.7 effect size (Cohen’s *d*) difference between treatment groups in the primary outcome (CVR to hypercapnia) at alpha = 0.05.

Using a per-protocol analysis and setting compliance to 80%, significant effects of treatment on mean changes in the variables (mood states, SMCs and QoL) between the groups were determined by a one-way MANOVA (IBM SPSS version 24, New York, NY, USA). Effect sizes are indicated by Cohen’s *d* or, if group sizes were different, by Hedge’s g. Using the 2 × 2 factorial design, effects of fish oil and curcumin treatment were also assessed independently with two-way ANOVA:

- Fish oil (FO and FO + CUR group) vs. no fish oil (CUR and PL group);
- Curcumin (CUR and FO + CUR group) vs. no curcumin (FO and PL group).

To examine the influence of APOE4 status on fish oil’s effects on mental wellbeing and QoL, an independent t test was used to determine differences in response to fish oil between APOE4 carriers and non-carriers. Additional post-hoc analyses (one-way MANOVA) were performed to look at treatment changes in variables between groups in APOE4 non-carriers only.

Pearson’s correlation analysis was used to determine whether changes in mood states and SMCs were related to changes in QoL. All results are presented as the mean ± standard error of mean (SEM). As this is an exploratory analysis, no adjustments were made for multiple comparisons.

## 3. Results

### 3.1. Participant Disposition and Baseline Characteristics

Of the 152 participants enrolled in this study between June 2017 and August 2018, 134 completed the intervention and 126 were compliant with supplementation (PL *n* = 32, FO *n* = 32, CUR *n* = 31, FO + CUR *n* = 31; for CONSORT diagram see Kuszewski et al. [24]). Four participants experienced side effects with supplementation (digestive problems: PL *n* = 1, CUR *n* = 1, FO + CUR *n* = 1; reflux: PL *n* = 1) and seven reported unrelated health issues, of which two occurred before supplementation was commenced. Of the remaining five health issues, two occurred in the fish oil group (pneumonia), two in the curcumin group (vein thrombosis, knee operation) and one in the combination group (heart attack). However, they were unlikely to be related to supplementation. For analysis of mental wellbeing measures, three participants had to be excluded: one participant was unable to complete the mental wellbeing questionnaires at week 16 (FO + CUR group), one participant experienced major life changes during the trial, affecting mental wellbeing measures (CUR group) and one participant had incomplete data (FO group), leaving 123 participants for the final analysis (PL *n* = 32, FO *n* = 31, CUR *n* = 30, FO + CUR *n* = 30).

Participants’ baseline characteristics are described in Table 1. Participants were, on average, elderly, marginally obese, had low total mood disturbance and their average CES-D score was 9.0 ± 0.6—well below the cut-off score of 16/60 for suspected depression. Furthermore, 72% (*n* = 109) of participants indicated that they have SMCs (first question of the SMCs questionnaire), with the total score of SMCs averaging 36 ± 2%. There were no significant differences in baseline characteristics between groups, except for the POMS mood subscale of tension (PL: 16.0 ± 2.2 vs. FO + CUR: 23.7 ± 3.1, *p* = 0.040).

### 3.2. Effects of Treatment on Mental Wellbeing Measures

Curcumin supplementation improved vigour compared to placebo (*p* = 0.044, Cohen’s *d* = 0.55). Supplementation with fish oil, alone or in combination with curcumin, for 16 weeks did not significantly affect mood states (Table 2).

SMCs were unaffected by fish oil supplementation, while curcumin and the combination of fish oil and curcumin supplementation tended to reduce SMCs. Combining these two groups in the 2 × 2 factorial analysis showed a 21% reduction in SMCs from baseline following curcumin supplementation, which was significant compared to no curcumin supplementation (CUR *n* = 60: −7.8 ± 2.0% vs. no CUR *n* = 63: −2.2 ± 1.7%, *p* = 0.038, Cohen’s *d* = 0.38). This reduction was even more significant among participants who reported SMCs at baseline (CUR *n* = 44: −9.4 ± 2.5% vs. no CUR *n* = 48: −2.4 ± 2.0%; *p* = 0.029, Cohen’s *d* = 0.46). The reduction in SMCs following curcumin supplementation (*n* = 60) was correlated with changes in confusion (R = 0.392, *p* = 0.002) and depression (R = 0.356, *p* = 0.006).

### 3.3. Effects of Treatment on Quality of Life

The overall score of QoL as well as the subcomponents of physical and mental wellbeing were not significantly affected by treatment (Table 2). However, the observed changes in vigour were correlated with changes in overall QoL (whole study population *n* = 122: R = 0.323, *p* < 0.001; curcumin group *n* = 59: R = 0.418, *p* = 0.001) and changes in SMCs were inversely correlated with changes in overall QoL (whole study population *n* = 122: R = −0.360, *p* < 0.001; curcumin group *n* = 59: R = −0.472, *p* < 0.001).

### 3.4. Influence of APOE4 Status

Of the 65 participants who were taking fish oil supplements, 26% were APOE4 carriers, with an average plasma APOE4 concentration of 76.7 ± 10.9 μg/mL. APOE4 carriers were slightly younger with a lower mean BMI but had the same percentage of self-reported depressive symptoms compared to APOE4 non-carriers. Baseline demographics or wellbeing measures were not significantly different between APOE4 carriers and non-carriers (Table 3).

**Figure 1:**
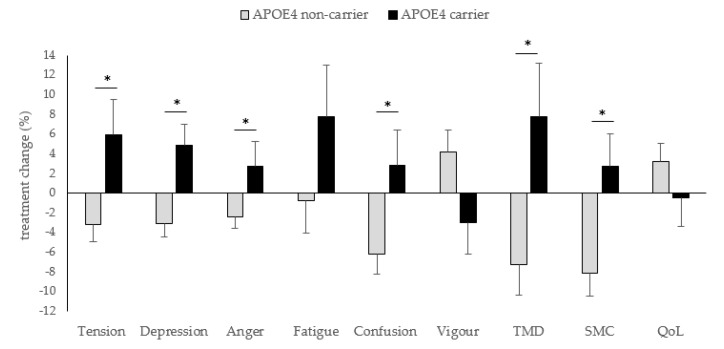
Differences between APOE4 carriers (*n* = 16) and APOE4 non-carriers (*n* = 44) in treatment changes of mental wellbeing measures and quality of life following fish oil supplementation. * Significant, *p* < 0.05. TMD: total mood disturbance, SMC: subjective memory complaints, QoL: quality of life.

APOE4 status significantly influenced effects of fish oil on mental wellbeing measures but not QoL. In general, while APOE4 carriers had a negative response to fish oil supplementation, i.e., greater mood disturbance and more SMCs, APOE4 non-carriers showed improvements in all mental wellbeing measures (Figure 1). Changes in tension (*p* = 0.014, Hedge’s g = 0.74), depression (*p* = 0.003, Hedge’s g = 0.90), anger (*p* = 0.043, Hedge’s g = 0.60), confusion (*p* = 0.028, Hedge’s g = 0.66), total mood disturbance (TMD) (*p* = 0.016, Hedge’s g = 0.72) and SMCs (*p* = 0.015, Hedge’s g = 0.75) following fish oil supplementation were significantly different between APOE4 carriers and non-carriers.

There were no differences between APOE4 carriers and non-carriers in erythrocyte EPA and DHA levels at baseline or in the changes in EPA (APOE4: 1.25 ± 0.32% vs. non-APOE4: 1.21 ± 0.47%, *p* = 0.756) and DHA (APOE4: 4.16 ± 1.33% vs. non-APOE4: 4.65 ± 1.39%, *p* = 0.256) levels following treatment.

### 3.5. Subanalysis in APOE4 Non-Carriers

Since APOE4 status influenced responses to fish oil supplementation, we re-examined the effect of fish oil supplementation in APOE4 non-carriers after excluding APOE4 carriers from the fish oil and fish oil + curcumin group (*n* = 17). This exploratory subanalysis showed a significant increase in vigour (*p* = 0.030, Hedge’s g = 0.56) and decrease in TMD (*p* = 0.048, Hedge’s g = 0.55) following fish oil supplementation compared to placebo (Table 4). The decrease in TMD following fish oil supplementation was inversely correlated with changes in vigour (R = −0.937, *p* < 0.001) and positively correlated with changes in fatigue (R = 0.590, *p* = 0.004). The combination of fish oil and curcumin significantly decreased SMCs (*p* = 0.029, Hedge’s g = 0.57).

The increase in overall QoL score following fish oil supplementation was not significant, but changes in vigour were correlated with changes in overall QoL (whole group *n* = 106: R = 0.287, *p* = 0.003; FO supplementation *n* = 44: R = 0.433, *p* = 0.003), and changes in TMD were inversely correlated with changes in overall QoL (R= −0.453, *p* < 0.001; FO supplementation *n* = 44: R= −0.597, *p* < 0.001).

## 4. Discussion

This exploratory analysis provides supportive evidence of curcumin’s mood-enhancing effects and furthermore shows curcumin’s potential to reduce SMCs in overweight or obese middle-aged and older adults without clinical depression. However, combining curcumin with fish oil did not result in any additional benefits on mental wellbeing. Fish oil supplementation alone did not affect mental wellbeing, although the response to fish oil was significantly affected by APOE4 status. APOE4 non-carriers showed improvements in mental wellbeing, whereas APOE4 carriers did not respond to fish oil. A subanalysis of the whole study cohort, excluding APOE4 carriers, revealed that fish oil supplementation improved vigour and decreased TMD.

QoL was not significantly improved, which might be due to the fact that baseline overall QoL scores were already relative high (average 69.9 ± 2%) and comparable to results from a large survey across Australian households [27], showing an average overall score of 71%. Nevertheless, improvements in vigour and SMCs following curcumin supplementation and improvements in vigour and TMD following fish oil supplementation were correlated with improved overall QoL, suggesting that curcumin’s and fish oil’s effects are clinically relevant.

### 4.1. Curcumin Supplementation

The observed mood-enhancing effect of curcumin, i.e., increase in vigour, is consistent with previous studies study by Cox et al., which found reductions in fatigue following 4 and 12 weeks of supplementation in older non-depressed adults [9,10]. Furthermore, Cox et al. found significant reductions in tension, anger, confusion and TMD following supplementation. However, these effects were only short-term and were not sustained after 12 weeks of supplementation [10]. In both studies, Cox et al. supplemented their participants with 400 mg Longvida curcumin (80 mg curcumin) once a day; however, we chose to give this dose twice daily to ensure a sustained level of curcumin in the blood. Consistent with findings from Cox et al. we observed reductions in confusion and TMD following 16 weeks of curcumin supplementation; however, they were not significant compared to placebo and combining curcumin with fish oil did not result in additional effects on these outcomes. Thus, it might be possible that curcumin has only short-term effects on confusion and TDM but stronger, longer-lasting effects on fatigue-vigour. Further studies are warranted to confirm the promising mood-enhancing effects of curcumin in older adults without clinical depression and identify the underlying mechanisms and how the efficacy of curcumin could be improved. Moreover, we found that the change in vigour was positively correlated with change in quality of life; however, further investigation is needed to determine whether the mood-enhancing effects of curcumin can help to prevent the onset of depression.

Next to improvements in vigour, we found that curcumin reduced SMCs, which—to the best of our knowledge—is the first evidence of this benefit. Increasing evidence links SMCs to increased dementia risk, suggesting that SMCs reflect early, subtle cognitive changes [7]. This is supported by neuroimaging studies, which indicate changes in brain structure and function in individuals with SMCs [33]. SMCs have also been shown to be closely related to poor mood and negatively impact QoL in healthy older adults [4,34]. The observed attenuation in SMCs following curcumin might thus be partly mediated by its mood-enhancing effects, which resulted in slight, non-significant reductions in depressive mood and confusion that were, however, significantly correlated with decreased SMCs. Additionally, our observation that the reduction in SMCs following curcumin was related to increased QoL is encouraging. Targeting SMCs might thus offer an early window of opportunity to intervene prior to the development of poor mood and the detection of measurable cognitive deficits.

Nevertheless, changes in SMCs during the 16 week intervention period and correlations with changes in mood states and QoL need to be interpreted with caution. The assessment of SMCs estimates SMCs over the last two years; therefore a longer intervention time is needed to meaningfully monitor changes in SMCs. Moreover, assessment of SMCs in a clinical setting has its limitations as it is difficult to distinguish between worried and well individuals and those who have subtle cognitive impairments [35]. Lastly, a further limitation is that the SMCs questionnaire utilized in this study, although more extensive than others with 27 items, has not been validated before in cognitively unimpaired elderly.

To the best of our knowledge, only two other studies have examined the potential of diet or dietary supplements to reduce SMCs. An observational study showed an inverse correlation between adherence to a healthy diet—mix of a Mediterranean diet and Dietary Approaches to Stop Hypertension (DASH) diet—and SMCs in adults aged above 70 years who did not suffer from depression [36]. Moreover, a 12 week clinical trial of BrainPower Advanced, a supplement containing a mix of 15 ingredients (Ginkgo biloba extract, green tea extract, l-pyroglutamic acid amongst others), showed improvements in SMCs compared to placebo in older adults (average 67 years) [37].

A healthy diet and dietary supplements, such as curcumin, might thus have the potential to attenuate SMCs. However, future studies are needed to confirm these encouraging findings, further investigate the long-term effects of reducing SMCs on cognitive function, mood and QoL and explore potential underlying mechanisms. These studies might also need to incorporate neuroimaging to more accurately assess SMCs in individuals at baseline.

### 4.2. Effects of Fish oil Supplementation Influenced by APOE4 Status

In line with two previous studies conducted in healthy older adults without clinical depression, fish oil supplementation did not significantly affect mental wellbeing measures [17,19]. However, we found that the effect of fish oil on mental wellbeing was significantly influenced by APOE4 status; subanalysis in APOE4 non-carriers showed that fish oil supplementation significantly improved vigour and reduced TMD. The reduction in TMD was mostly driven by changes in vigour and fatigue. Improvements in these mood subscales might increase motivation to be more proactive in making healthy lifestyle choices such as eating a more healthy diet, which in turn can further improve mood and mental wellbeing [38,39].

Our finding that APOE4 status affects the response to fish oil supplementation is consistent with previous literature demonstrating a fish oil–APOE4 interaction [20,22]. For instance, APOE4 carriers were shown to be less sensitive to the protective effects of fish oil/consumption of fatty fish on cognitive function and all-cause dementia risk [40,41]. The mechanisms underlying the differential responses to fish oil are, however, poorly understood and need further investigation.

### 4.3. Combination of Fish Oil and Curcumin

The combination of fish oil and curcumin had neither an additive nor synergistic influence on mental wellbeing and QoL. Since this is the first exploratory analysis to look at the effects of this nutrient combination on mental wellbeing, further investigations are needed to identify whether there are potential synergistic effects between LCn-3PUFAs and curcumin in humans and whether they might depend on an optimal dose combination of fish oil and curcumin or might only benefit certain population groups.

### 4.4. Study Limitations

As this was an exploratory analysis of secondary outcomes of a large interventions study, it was not powered to detect differences in changes in mental wellbeing measures and QoL.

Another limitation is that APOE4 status was determined by measuring APOE4 in plasma, which is not as informative as APOE4 genotyping. It only indicates that a participant has the APOE4 allele, but not how many copies. Therefore, the exact genotype remains unknown. Our finding that the response to fish oil on mental wellbeing measures is dependent on APOE4 status is preliminary; further studies are needed to confirm this finding and whether it can be extrapolated to other populations.

## 5. Conclusions

This exploratory analysis of a 16 week dietary intervention trial in overweight or obese middle-aged and older adults without clinical depression adds further evidence that curcumin supplementation has potential beneficial effects on mood. Furthermore, our findings indicate that curcumin supplementation can reduce SMCs and that the improvements in both mood and SMCs are associated with improved QoL. These potential benefits of curcumin warrant further evaluation. Combining curcumin with fish oil did not result in additional benefits. Fish oil independently improved vigour and total mood disturbance, but only in APOE4 non-carriers. The observation that the mental wellbeing response to fish oil was influenced by APOE4 status should be followed up with studies designed to compare effects of fish oil on mental wellbeing between APOE4 carriers and non-carriers.

## Figures and Tables

**Table 1:** Participants’ baseline characteristics per group.

Characteristics	PL (*n* = 36)	FO (*n* = 39)	CUR (*n* = 38)	FO + CUR (*n* = 39)
Sex (female %)	50	56	55	56
Age (years)	65.4 ± 1.3	65.4 ± 1.2	65.4 ± 1.2	66.2 ± 1.3
BMI (kg/m^2^)	31.0 ± 0.7	31.0 ± 0.7	30.5 ± 0.7	30.9 ± 0.6
Depressive symptoms (%)	13.4 ± 1.9	14.0 ± 1.7	17.0 ± 2.1	15.4 ± 2.4
**Mood states (POMS)**				
Tension (%)	16.0 ± 2.2	16.8 ± 2.2	23.1 ± 2.9	23.7 ± 3.1 *
Depression (%)	5.6 ± 1.1	7.9 ± 1.8	9.6 ± 1.9	10.6 ± 2.1
Anger (%)	7.0 ± 1.0	7.1 ± 1.4	10.7 ± 2.0	10.5 ± 1.7
Fatigue (%)	28.1 ± 4.1	28.1 ± 4.6	30.4 ± 3.9	28.3 ± 3.5
Confusion (%)	23.8 ± 2.9	23.9 ± 3.1	28.6 ± 3.3	24.5 ± 2.8
Vigour (%)	56.2 ± 2.8	50.5 ± 3.0	49.2 ± 2.8	52.5 ± 3.5
TMD ^a^ (%)	−40.1 ± 3.6	−33.7 ± 4.6	−28.7 ± 4.2	−33.0 ± 5.0
Subjective memory complaints (%)	37.5 ± 4.1	33.7 ± 4.1	35.7 ± 3.9	39.4 ± 4.5
Quality of Life (%)	70.4 ± 2.4	72.5 ± 1.9	67.1 ± 2.5	69.4 ± 2.4

Values are expressed as the mean ± SEM. ^a^ Greater negative value equals better overall mood. * Significant compared to placebo, *p* < 0.05. BMI, body mass index; CUR, curcumin alone; FO, fish oil alone; FO + CUR, fish oil and curcumin combination; PL, placebo; TMD, total mood disturbance.

**Table 2:** Treatment changes in measures of mental wellbeing and quality of life (*n* = 123).

	PL (*n* = 32)	FO (*n* = 31)	CUR (*n* = 30)	FO + CUR (*n* = 30)
Tension (%)	0.4 ± 1.5	−0.5 ± 2.1	0.0 ± 2.1	−0.8 ± 2.5
Depression (%)	−0.6 ± 1.3	−1.6 ± 1.8	−1.8 ± 2.1	−0.1 ± 1.6
Anger (%)	−2.1 ± 1.2	0.2 ± 1.5	−2.1 ± 2.1	−2.2 ± 1.7
Fatigue (%)	−3.1 ± 4.0	0.1 ± 4.0	−1.5 ± 3.5	3.8 ± 3.9
Confusion (%)	−3.1 ± 2.4	−3.4 ± 2.8	−7.6 ± 3.1	−3.8 ± 2.4
Vigour (%)	−3.7 ± 2.6	1.5 ± 2.9	3.5 ± 2.0 *	2.5 ± 2.4
TMD ^a^ (%)	2.0 ± 3.8	−2.6 ± 4.2	−6.1 ± 3.3	−3.1 ± 3.7
Subjective memory complaints (%)	−1.8 ± 2.5	−2.6 ± 2.4	−6.7 ± 2.7	−8.9 ± 3.1
Quality of Life (%)	0.3 ± 1.8	2.4 ± 1.8	2.0 ± 2.1	2.1 ± 2.4

Values are expressed as the mean ± SEM. ^a^ Greater reduction is favourable. * Significant compared to placebo, *p* < 0.05. BMI, body mass index; CUR, curcumin alone; FO, fish oil alone; FO + CUR, fish oil and curcumin combination; PL, placebo; TMD, total mood disturbance.

**Table 3:** Baseline demographics and wellbeing measures in APOE4 carriers vs. non-carriers.

	APOE4 Non-Carrier (*n* = 48)	APOE4 Carrier (*n* = 17)	*p*-Value
Sex (female %)	52	53	
Age (years)	66.3 ± 1.2	64.0 ± 1.8	0.298
BMI (kg/m^2^)	31.5 ± 0.6	29.3 ± 0.7	0.052
Depressive symptoms (%)	15.2 ± 1.8	15.4 ± 4.2	0.953
EPA (%)	1.06 ± 0.37	1.04 ± 0.44	0.807
DHA (%)	5.49 ± 1.36	5.73 ± 1.31	0.527
Tension (%)	20.9 ± 2.3	25.5 ± 5.3	0.356
Depression (%)	10.4 ± 1.8	9.7 ± 3.4	0.855
Anger (%)	8.5 ± 1.4	12.2 ± 3.1	0.214
Fatigue (%)	30.3 ± 4.1	23.3 ± 3.9	0.219
Confusion (%)	26.8 ± 2.8	22.3 ± 4.0	0.388
Vigour (%)	49.4 ± 2.7	52.2 ± 5.7	0.619
TMD ^a^ (%)	−30.0 ± 4.2	−33.6 ± 8.0	0.671
Subjective memory complaints (%)	41.9 ± 3.8	30.6 ± 6.5	0.137
Quality of life (%)	69.5 ± 2.0	72.7 ± 3.4	0.430

Values are expressed as the mean ± SEM. ^a^ Greater negative value equals better overall mood. APOE4, apolipoprotein E4; BMI, body mass index; DHA, docosahexaenoic acid; EPA, eicosapentaenoic acid; TMD, total mood disturbance.

**Table 4:** Subanalysis of treatment changes in measures of mental wellbeing and quality of life in APOE4 non-carriers (*n* = 106).

	PL (*n* = 32)	FO (*n* = 22)	CUR (*n* = 30)	FO + CUR (*n* = 22)
Tension (%)	0.4 ± 1.5	−3.6 ± 1.8	0.0 ± 2.1	−2.7 ± 3.0
Depression (%)	−0.6 ± 1.3	−4.4 ± 1.9	−1.8 ± 2.1	−1.7 ± 1.9
Anger (%)	−2.1 ± 1.2	−1.8 ± 1.3	−2.1 ± 2.1	−2.9 ± 2.0
Fatigue (%)	−3.1 ± 4.0	−3.8 ± 4.4	−1.5 ± 3.5	2.3 ± 4.8
Confusion (%)	−3.1 ± 2.4	−7.9 ± 2.7	−7.6 ± 3.1	−4.4 ± 3.1
Vigour (%)	−3.7 ± 2.6	4.8 ± 3.3 *	3.5 ± 2.0 *	3.6 ± 3.1
TMD ^a^ (%)	2.0 ± 3.8	−9.1 ± 4.0 *	−6.1 ± 3.3	−5.4 ± 4.8
Subjective memory complaints (%)	−1.8 ± 2.5	−6.1 ± 2.6	−6.7 ± 2.7	−11.0 ± 3.9 *
Quality of life (%)	0.3 ± 1.8	4.6 ± 2.0	2.0 ± 2.1	1.8 ± 2.9

Values are expressed as the mean ± SEM. ^a^ greater reduction being favourable. * Significant compared to placebo, *p* < 0.05. CUR, curcumin alone; FO, fish oil alone; FO + CUR, fish oil and curcumin combination; PL, placebo; TMD, total mood disturbance.

## References

[1] Farioli-Vecchioli S., Sacchetti S., di Robilant N.V., Cutuli D. (2018). The Role of Physical Exercise and Omega-3 Fatty Acids in Depressive Illness in the Elderly. Curr. Neuropharmacol..

[2] Barua A., Ghosh M.K., Kar N., Basilio M.A. (2011). Prevalence of depressive disorders in the elderly. Ann. Saudi Med..

[3] Okereke O.I., Reynolds C.F., Mischoulon D., Chang G., Cook N.R., Copeland T., Friedenberg G., Buring J.E., Manson J.E. (2018). The VITamin D and OmegA-3 TriaL-Depression Endpoint Prevention (VITAL-DEP): Rationale and design of a large-scale ancillary study evaluating vitamin D and marine omega-3 fatty acid supplements for prevention of late-life depression. Contemp. Clin. Trials.

[4] Balash Y., Mordechovich M., Shabtai H., Giladi N., Gurevich T., Korczyn A.D. (2013). Subjective memory complaints in elders: Depression, anxiety, or cognitive decline?. Acta Neurol. Scand..

[5] Glodzik-Sobanska L., Reisberg B., De Santi S., Babb J.S., Pirraglia E., Rich K.E., Brys M., de Leon M.J. (2007). Subjective memory complaints: Presence, severity and future outcome in normal older subjects. Dement. Geriatr. Cogn. Disord..

[6] Jessen F., Wiese B., Bachmann C., Eifflaender-Gorfer S., Haller F., Kölsch H., Luck T., Mösch E., van den Bussche H., Wagner M. (2010). Prediction of dementia by subjective memory impairment: Effects of severity and temporal association with cognitive impairment. Arch. Gen. Psychiatry.

[7] Kaup A.R., Nettiksimmons J., LeBlanc E.S., Yaffe K. (2015). Memory complaints and risk of cognitive impairment after nearly 2 decades among older women. Neurology.

[8] Fusar-Poli L., Vozza L., Gabbiadini A., Vanella A., Concas I., Tinacci S., Petralia A., Signorelli M.S., Aguglia E. (2020). Curcumin for depression: A meta-analysis. Crit. Rev. Food Sci. Nutr..

[9] Cox K.H., Pipingas A., Scholey A.B. (2015). Investigation of the effects of solid lipid curcumin on cognition and mood in a healthy older population. J. Psychopharmacol..

[10] Cox K.H.M., White D.J., Pipingas A., Poorun K., Scholey A. (2020). Further Evidence of Benefits to Mood and Working Memory from Lipidated Curcumin in Healthy Older People: A 12-Week, Double-Blind, Placebo-Controlled, Partial Replication Study. Nutrients.

[11] Abdolahi M., Sarraf P., Javanbakht M.H., Honarvar N.M., Hatami M., Soveyd N., Tafakhori A., Sedighiyan M., Djalali M., Jafarieh A. (2018). A Novel Combination of ω-3 Fatty Acids and Nano-Curcumin Modulates Interleukin-6 Gene Expression and High Sensitivity C-reactive Protein Serum Levels in Patients with Migraine: A Randomized Clinical Trial Study. CNS Neurol. Disord. Drug Targets.

[12] Larrieu T., Layé S. (2018). Food for Mood: Relevance of Nutritional Omega-3 Fatty Acids for Depression and Anxiety. Front. Physiol..

[13] Yang Y., Kim Y., Je Y. (2018). Fish consumption and risk of depression: Epidemiological evidence from prospective studies. Asia Pac. Psychiatry.

[14] Bae J.H., Kim G. (2018). Systematic review and meta-analysis of omega-3-fatty acids in elderly patients with depression. Nutr. Res..

[15] Grenyer B.F., Crowe T., Meyer B., Owen A.J., Grigonis-Deane E.M., Caputi P., Howe P.R. (2007). Fish oil supplementation in the treatment of major depression: A randomised double-blind placebo-controlled trial. Prog. Neuropsychopharmacol. Biol. Psychiatry.

[16] Meyer B.J., Grenyer B.F., Crowe T., Owen A.J., Grigonis-Deane E.M., Howe P.R. (2013). Improvement of major depression is associated with increased erythrocyte DHA. Lipids.

[17] Giltay E.J., Geleijnse J.M., Kromhout D. (2011). Effects of n-3 fatty acids on depressive symptoms and dispositional optimism after myocardial infarction. Am. J. Clin. Nutr..

[18] Sinn N., Milte C.M., Street S.J., Buckley J.D., Coates A.M., Petkov J., Howe P.R. (2012). Effects of n-3 fatty acids, EPA v. DHA, on depressive symptoms, quality of life, memory and executive function in older adults with mild cognitive impairment: A 6-month randomised controlled trial. Br. J. Nutr..

[19] Van de Rest O., Geleijnse J.M., Kok F.J., van Staveren W.A., Hoefnagels W.H., Beekman A.T., de Groot L.C. (2008). Effect of fish-oil supplementation on mental well-being in older subjects: A randomized, double-blind, placebo-controlled trial. Am. J. Clin. Nutr..

[20] Minihane A.M. (2016). Impact of Genotype on EPA and DHA Status and Responsiveness to Increased Intakes. Nutrients.

[21] Minihane A.M., Khan S., Leigh-Firbank E.C., Talmud P., Wright J.W., Murphy M.C., Griffin B.A., Williams C.M. (2000). ApoE polymorphism and fish oil supplementation in subjects with an atherogenic lipoprotein phenotype. Arter. Thromb. Vasc. Biol..

[22] Pontifex M., Vauzour D., Minihane A.M. (2018). The effect of APOE genotype on Alzheimer’s disease risk is influenced by sex and docosahexaenoic acid status. Neurobiol. Aging.

[23] Small G.W., Siddarth P., Li Z., Miller K.J., Ercoli L., Emerson N.D., Martinez J., Wong K.P., Liu J., Merrill D.A. (2018). Memory and Brain Amyloid and Tau Effects of a Bioavailable Form of Curcumin in Non-Demented Adults: A Double-Blind, Placebo-Controlled 18-Month Trial. Am. J. Geriatr. Psychiatry.

[24] Kuszewski J.C., Wong R.H.X., Wood L.G., Howe P.R.C. (2020). Effects of fish oil and curcumin supplementation on cerebrovascular function in older adults: A randomized controlled trial. Nutr. Metab. Cardiovasc. Dis..

[25] Radloff L.S. (1977). The CES-D Scale: A self-report depression scale for research in the general population. Appl. Psychol. Meas..

[26] Wyrwich K.W., Yu H. (2011). Validation of POMS questionnaire in postmenopausal women. Qual. Life Res..

[27] Butterworth P., Crosier T. (2004). The validity of the SF-36 in an Australian National Household Survey: Demonstrating the applicability of the Household Income and Labour Dynamics in Australia (HILDA) Survey to examination of health inequalities. BMC Public Health.

[28] Mishra G., Schofield M.J. (1998). Norms for the physical and mental health component summary scores of the SF-36 for young, middle-aged and older Australian women. Qual. Life Res..

[29] Altman D.G., Bland J.M. (2005). Treatment allocation by minimisation. BMJ.

[30] Gota V.S., Maru G.B., Soni T.G., Gandhi T.R., Kochar N., Agarwal M.G. (2010). Safety and pharmacokinetics of a solid lipid curcumin particle formulation in osteosarcoma patients and healthy volunteers. J. Agric. Food Chem..

[31] Cassiday L. (2016). Sink or swim: Fish oil supplements and human health. INFORM.

[32] Luo X.D., Feng J.S., Yang Z., Huang Q.T., Lin J.D., Yang B., Su K.P., Pan J.Y. (2020). High-dose omega-3 polyunsaturated fatty acid supplementation might be more superior than low-dose for major depressive disorder in early therapy period: A network meta-analysis. BMC Psychiatry.

[33] Hafkemeijer A., Altmann-Schneider I., Oleksik A.M., van de Wiel L., Middelkoop H.A., van Buchem M.A., van der Grond J., Rombouts S.A. (2013). Increased functional connectivity and brain atrophy in elderly with subjective memory complaints. Brain Connect..

[34] Mol M., Carpay M., Ramakers I., Rozendaal N., Verhey F., Jolles J. (2007). The effect of perceived forgetfulness on quality of life in older adults; a qualitative review. Int. J. Geriatr. Psychiatry.

[35] Ahmed S., Mitchell J., Arnold R., Dawson K., Nestor P.J., Hodges J.R. (2008). Memory complaints in mild cognitive impairment, worried well, and semantic dementia patients. Alzheimer Dis. Assoc. Disord..

[36] Adjibade M., Assmann K.E., Julia C., Galan P., Hercberg S., Kesse-Guyot E. (2019). Prospective association between adherence to the MIND diet and subjective memory complaints in the French NutriNet-Santé cohort. J. Neurol..

[37] Zhu J., Shi R., Chen S., Dai L., Shen T., Feng Y., Gu P., Shariff M., Nguyen T., Ye Y. (2016). The Relieving Effects of BrainPower Advanced, a Dietary Supplement, in Older Adults with Subjective Memory Complaints: A Randomized, Double-Blind, Placebo-Controlled Trial. Evid. Based Complement. Altern. Med..

[38] Firth J., Gangwisch J.E., Borisini A., Wootton R.E., Mayer E.A. (2020). Food and mood: How do diet and nutrition affect mental wellbeing?. BMJ.

[39] Gardner M.P., Wansink B., Kim J., Park S. (2014). Better moods for better eating? How mood influences food choice. J. Consum. Psychol..

[40] Huang T.L., Zandi P.P., Tucker K.L., Fitzpatrick A.L., Kuller L.H., Fried L.P., Burke G.L., Carlson M.C. (2005). Benefits of fatty fish on dementia risk are stronger for those without APOE epsilon4. Neurology.

[41] Whalley L.J., Deary I.J., Starr J.M., Wahle K.W., Rance K.A., Bourne V.J., Fox H.C. (2008). n-3 Fatty acid erythrocyte membrane content, APOE varepsilon4, and cognitive variation: An observational follow-up study in late adulthood. Am. J. Clin. Nutr..

